# Correction to “Incisional negative pressure wound therapy for the prevention of surgical site complications in paediatric patients with non‐idiopathic scoliosis: A randomized clinical trial”

**DOI:** 10.1111/iwj.70067

**Published:** 2024-10-01

Pérez‐Acevedo G, Torra‐Bou JE, Peiro‐García A, et al. Incisional negative pressure wound therapy for the prevention of surgical site complications in paediatric patients with non‐idiopathic scoliosis: a randomized clinical trial. *Int Wound J*. 2024; 21(9): e70034. doi:10.1111/iwj.70034

The following affiliations are incorrect:

2 Doctoral Program, Faculty of Nursing and Physiotherapy‐GESEC, University of Lleida, Barcelona, Spain

3 Researcher and GRECS‐IRBLleida, Tr2Lab (Tissue Repair and Regeneration Laboratory) Research Group, Institute for Research and Innovation in Life and Health Sciences in Central Catalonia (IRIS‐CC), Barcelona, Spain

4 Unidad de Columna, Hospital Sant Joan de Déu, Barcelona, Spain

It should have been:

2 Doctoral Program, Tissue Repair and Regeneration Laboratory (TR2Lab), Nursing and Physiotherapy Department, University of Lleida, Barcelona, Spain.

3 Institute for Research and Innovation in Life and Health Sciences in Central Catalonia (IRIS‐CC), Barcelona, Spain

4 Spine Unit, Sant Joan de Déu Hospital, Barcelona, Spain

The authors, Gemma Pérez‐Acevedo and Alejandro Peiró‐García, are incorrect. It should have been Gemma Perez‐Acevedo and Alejandro Peiro‐Garcia.

The ORCID is missing for the following authors:

Gemma Perez‐Acevedo: 0000‐0001‐8742‐5201

Alejandro Peiro‐Garcia: 0000‐0003‐2109‐8978

Inmaculada Vilalta‐Vidal: 0000‐0002‐7236‐3746

Mireia Urrea‐Ayala: 0000‐0002‐9271‐7468

Joan Blanco‐Blanco: 0000‐0002‐4868‐2974

In line 9 of the Results section, “One patient also excluded because of manipulation” is incorrect. It should have been: “One patient was also excluded because of manipulation.”

We apologize for these errors.

